# Treating equivalent cases differently: A comparative analysis of substance use disorder and type 2 diabetes in Norwegian treatment guidelines

**DOI:** 10.1111/jep.13693

**Published:** 2022-04-28

**Authors:** Fredrik D. Moe, Henrik Berg

**Affiliations:** ^1^ Department of Social Studies, Faculty of Social Sciences University of Stavanger Stavanger Norway; ^2^ Centre for Alcohol and Drug Research Stavanger University Hospital Stavanger Norway; ^3^ Department of Clinical Psychology, Faculty of Psychology University of Bergen Bergen Norway; ^4^ Centre for Sustainable Healthcare Education, Faculty of Medicine University of Oslo Oslo Norway; ^5^ Faculty of Health and Sport Sciences University of Agder Kristiansand Norway

**Keywords:** chronic illness, global policymaking, substance use disorder, treatment guidelines, type 2 diabetes

## Abstract

**Background:**

Substance use disorder (SUD) is often understood as a chronic illness. **Aims**: This paper investigates whether SUD is treated as a chronic illness.

**Method:**

To this aim, we have used World Health Organizations (WHO's) definition of chronic illness to conduct a comparative analysis of SUD and type 2 diabetes (T2D), which is another chronic illness.

**Results:**

When analysing Norwegian treatment guidelines, we found that only the T2D guideline reflects the WHO's conceptualization of chronic illnesses. We argue that this discrepancy implies that SUD is understood as a moral and legal problem, while T2D is conceptualized as a somatic illness. We discuss how social, political and historical conditions of the possibility for understanding SUD are interwoven with normative presumptions about the clinician, patient, treatment guidelines and drug policies in a way that may impede the development of continuing care.

**Conclusion:**

The paper concludes that the delivery of treatment services is inequitable as SUD is not treated as a chronic illness.

## 1 INTRODUCTION

Professional healthcare institutions vary in terms of the number of diseases they define as chronic.^1^ While the World Health Organization (WHO) defines cardiovascular diseases, cancers, chronic respiratory diseases and diabetes as chronic illnesses, the Centres for Disease Control and Prevention (CDC) omits chronic respiratory diseases.^1^ Furthermore, the temporal criterion, describing the duration of symptoms in chronic illness, varies. MedicineNet states that chronic illnesses last 3 months or longer,^2^ while the CDC states that they last for 1 year or more.^3^ WHO states that ‘chronic diseases,[sic] tend to be of long duration and are the result of a combination of genetic, physiological, environmental and behavioural factors’.^4^ Thus, a main characteristic of chronic illnesses is their relatively long duration. However, chronic illness is also associated with functional impairment, relapse risk and other health complications.^1^ All of these may occur in illnesses such as cancer, diabetes and substance use disorder. In this analysis, ‘illness’ refers to an extensive disease construction which includes social, psychological, behavioural and biological factors.^5^ Thus, a chronic illness involves a complex web of social, psychological, behavioural and biological factors with a relatively long duration.

Substance use disorder (SUD) and type 2 diabetes (T2D) are different illnesses and thus warrant different treatment components. However, according to WHO's criteria, they overlap in terms of chronic illness development. Both are influenced by genetic, psychosocial, behavioural and environmental factors and require sustained behaviour change to achieve recovery. In this comparative analysis, we use the following three research questions to analyse SUD and T2D as *chronic illnesses*:

(1) Are SUD and T2D conceptualized as chronic illnesses?

(2) Are SUD and T2D treated as chronic illnesses in practice?

(3) How can we explain any discrepancies in the treatment of these illnesses which is not found in the conceptualization?

## 2 METHOD

Treatment guidelines are official state documents that inform practice. The guidelines are typically open access and produced by an institution, such as the Norwegian Directorate of Health (NDH). The NDH was created to improve the quality of health services and promote better public health.^6^ Hence, the guidelines are produced with the purpose of informing practice and are a means of communicating with healthcare professionals. The Norwegian treatment guidelines for SUD and T2D constitute the corpus of documents in the present comparative analysis. However, the two guidelines refer to other documents, such as research studies, clinical experience, service‐user perspectives and legal documents.^6^ These guidelines have been purposely selected as they contain the relevant treatment recommendations that practitioners are asked to follow.^7^ The purpose of selecting these cases is to investigate the extent to which SUD and T2D are treated as chronic illnesses.

## 3 ARE T2D AND SUD CONCEPTUALIZED AS CHRONIC ILLNESSES?

To examine whether T2D and SUD are conceptualized as chronic illnesses, we analyse them according to the WHO definition of chronic illness. Thus, T2D and SUD should be seen as long‐lasting and the result of a combination of behavioural, physiological, genetic and environmental factors.^4^

### 3.1 T2D

Research shows that genetic factors influence the development of T2D.^8^ A study of monozygotic twins found that the genetic heritability of T2D was approximately 40%,^9^ while other studies have found an estimated heritability of 72%.^10^ Generally, studies report that the range of genetic heritability varies from 45% to 75%.^11^ A main physiological component of T2D is hypoglycaemia, which is an abnormally low level of glucose in the blood.^12^ However, research has not been able to explain the underlying pathophysiological mechanisms for hypoglycaemia.^13^ Although genetic heritability is a significant factor for developing T2D, environmental factors such as diet and lifestyle are catalysts. For example, a sedentary lifestyle and a high caloric diet are associated with developing T2D.^14^ Hence, genetics and the environment interact in disease development.^15^ Research on T2D and specific changes in diet and exercise has shown the latter to reduce the severity of disease and effectively control T2D.^16^ Changes in diet and activity levels have increased the probability of remission, despite ongoing T2D illness.^17^ Therefore, individuals with T2D can change behaviour to control, or at least reduce, disease progression. Treatment of T2D focuses on lifestyle changes, particularly with regard to diet, exercise and stress management, as well as on medication.^18^, ^19^

### 3.2 Substance use disorder

Research shows that genetic factors influence the development of SUD.^20^, ^21^ There is a higher rate of heritability among monozygotic than dizygotic twins.^22^, ^23^ Physiological factors also play a crucial role in sustaining, and probably in developing, SUD. Much discussion revolves around how certain substances induce pleasurable effects by affecting neurotransmitters, such as dopamine.^24^ Repeated administration of an addictive substance leads to increased tolerance, and ceasing to take the substance leads to withdrawal symptoms.^25^ Neuroscientific research suggests that long‐term regular use of addictive psychoactive substances can influence the brain's reward system.^25^ However, the development of SUD is also based on environmental factors. The degree of exposure and access to addictive substances, exposure to community disorder or disadvantage and difficulty accessing treatment, all influence the development of SUD and increase the frequency of substance use.^26^, ^27^

Another aetiological factor in SUD is personal contribution. Research indicates that personal contribution interacts with social determinants and genetics, which also applies for a number of chronic diseases, such as hypertension. Inherited salt sensitivity is a possible precipitant of the development of one type of hypertension among males. The total risk of developing hypertension, however, depends on familial salt use patterns, personal contribution, and genetics.^28^ Therefore, inherited salt sensitivity is a genetic risk factor among other risk factors in hypertension development. The case of hypertension development illustrates how different risk factors contribute to illness development, which appears equally relevant to how SUD develops.

As mentioned above, the minimum symptom duration required in order for an illness to be classified as chronic ranges from 3 to 12 months.^1^, ^2^ WHO does not state explicitly the minimum symptom duration but states that the illness must be of a long duration.^4^ The diagnostic assessment of SUD states that one must exhibit at least 2 of the 11 diagnostic criteria within a 12‐month period to qualify for an SUD diagnosis.^29^ Thus, the SUD diagnosis fulfils the temporal criterion in all of the abovementioned definitions of chronic illness. Additionally, SUD and T2D fulfil WHO's criteria for chronic illness. Thus, they are comparable in terms of chronicity.

## 4 ARE SUD AND T2D TREATED AS CHRONIC ILLNESSES IN PRACTICE?

### 4.1 Treatment of T2D

The T2D guideline states that treatment of T2D varies depending on the individual. Often, treatment consists of continuing care and planned follow‐up.^30^ Typical components are self‐monitoring and self‐management of blood glucose by means of diet, exercise and medication.^31^ Individuals at high risk of developing T2D can prevent the disease if they make lifestyle changes.^18^ These recommendations are reflected in the T2D guideline, which states that patients in stable phases should have ‘[…] 2‐4 consultations a year with GP/nurse. At least one of the consultations should be at the GP and contain a broad check of the patient's diabetes (annual control)’.^32^ Furthermore, ‘[i]t is recommended that people with T2D are physically active, with moderate to high‐intensity activity for a minimum of 150 minutes per week, distributed over at least three days and with no more than two consecutive days without physical activity’,^32^ in addition to making dietary changes.^32^ Moreover, the guideline states that ‘the GP should refer the patient to a Beginner's (sic) course’^32^ and that ‘in addition to being legally entitled, it is well‐documented that a good and basic education in one's disease (especially in the case of chronic, life‐long illnesses) reduces adverse outcomes […], improves functioning and strengthens self‐efficacy’.^32^ The guideline also specifies that the GP should conduct an annual check using standardized procedures.^32^

The T2D guideline also refers to the duration of treatment, essential aspects of the disease, and annual screenings. The guideline recommends a minimum of one GP consultation per year.^32^ Furthermore, it recommends that patients in stable phases have two to four consultations per year with their GP or nurse.^32^ The recommendation for patients with well‐regulated diabetes is one additional check‐up in addition to the annual check.^32^

If the treatment for stable patients is insufficient, the guideline recommends treatment every 3 months. T2D patients have follow‐up and treatment mainly with the GP.^32^ However, T2D patients with poor regulation, severe complications or other additional complicating diseases will also receive follow‐up from specialist health services. The responsibility is shared by the GP and the hospital, and good communication and collaboration are essential.^32^ It is recommended that T2D patients with poor regulation also receive assistance from diabetic teams in the specialist health service.^32^ These teams should include a senior consultant diabetologist, a nurse specializing in diabetes and a nutritionist.^32^ The guideline also mentions other professional groups, such as psychologists and social workers, as well as contact with a foot care clinic, and highlights the diabetic teams' primary assignments. The guideline clearly indicates the number and duration of follow‐ups, the involvement of treatment personnel and the division of responsibilities.

### 4.2 Treatment of substance use disorder

The treatment guideline for SUD contains three phases.^33^ These three phases are: (1) stabilization and detoxification, (2) inpatient treatment and (3) outpatient treatment. The SUD guideline focuses on the individual's treatment needs: ‘Treatment and follow‐up duration should be tailored to the individual's needs*’* ^33^ and ‘[i]f physical exercise/training therapy is desired as a part of treatment, this should be accommodated’.^33^ However, individual tailoring affects the clarity of the guideline. For example, the guideline does not specify a recommended period of follow‐up: ‘The total treatment duration must be tailored to the individual's need and may last for several years in the case of people with the most severe dependency issues. However, this does not mean years of inpatient treatment, but tailored forms of treatment’.^33^ Instead of prolonged treatment in inpatient institutions, tailored treatment includes user‐controlled admissions to inpatient facilities, outpatient treatment and municipal services.^33^ The guideline neither specifies the duration nor the number of follow‐ups; rather, it states that prolonged treatment is needed for some but not all patients.^33^ While the focus on tailoring follow‐up to accommodate individual needs is legitimate considering the variation in the duration of SUD,^34^ a disadvantage to this approach is that the guideline does not provide a reference point for treatment.

In the SUD guideline, long‐term treatment seems to be the responsibility of the municipality. However, there is a lack of specificity in the detailed information as to who is involved and what their responsibilities are. For example, there are specific recommendations regarding interventions for adolescents at risk of developing SUD. The SUD guideline focuses mainly on the usefulness of early interventions, advocating an ambulatory approach, appropriate diagnostic tools and family therapy.^33^ However, it does not elaborate on the intervention's organization, and details regarding responsibility (i.e., who does what when) are less specific. For example, the SUD guideline states that the school and the school nurse have responsibilities, but it neither mentions how the responsibility should be organized nor what measures to include. Likewise, the ambulatory approach lacks specificity concerning whom this may include and how it should be organized. Although the guideline mentions municipal involvement, this is somewhat vague: It states that the patient is entitled (by law) to an individual plan (IP) that coordinates their treatment.^33^ However, the specific procedure for implementing the IP is elusive: although it appears that this is something the service providers should do, the practical procedures of implementing the IP are unclear.

Two hallmarks of treating chronic illness are planned and regular follow‐ups, and systematic assessments.^35^, ^36^ The SUD guideline states that follow‐up and systematic assessment are essential. However, it does not indicate neither the recommended duration nor the number of follow‐ups. The lack of a point of departure means that it may be challenging for treatment professionals to implement planned and regular follow‐ups and systematic assessments. Thus, treatment services may lack the organizational foundation to deliver continuing care.

To summarize, the treatment guidelines for SUD and T2D differ in their specificity of treatment organization concerning chronic illness treatment principles. The T2D guideline emphasizes more precise treatment measures and thus appears easier to implement in treatment practice compared to the SUD guideline. For example, whereas the T2D guideline states a minimum number of follow‐ups, the SUD guideline does not, making it challenging for SUD patients and service providers to know how many times they should meet. This difference in the level of detail in the guidelines may provide one explanation as to why SUD is not treated as a chronic illness in practice while T2D is.

## 5 DISCUSSION

Our analysis suggests that SUD and T2D are conceptualized as chronic illnesses in accordance with WHO's definition. Our finding is on par with McLellan et al.^28^ who conclude that drug dependence is similar to other chronic illnesses, such as T2D, in terms of vulnerability, course and onset. Moreover, we have analysed the Norwegian treatment guidelines for SUD and T2D to investigate whether they are treated as chronic illnesses in practice. We found that both guidelines focus on long‐term treatment and systematic assessments, but that they differ in their level of detail. Our analysis finds that the T2D guideline is more explicit than the SUD guideline in its recommendations regarding the duration and number of follow‐ups and systematic assessment. Thus, we argue that the T2D guideline is less ambiguous and hence may be easier to translate into treatment practice compared to the SUD guideline. Although both guidelines underscore continuing treatment, the SUD guideline appears to fall short due to its general and imprecise recommendations. As a result, SUD may not be treated as a chronic illness in practice. Although SUD is increasingly understood as a chronic illness, few changes have been made in the social policies of dependence, research strategies used to evaluate outcome and clinical practice.^37^ Our analysis suggests that this is still true of SUD 22 years after the publication of McLellan et al.^28^

### 5.1 Historical similarities and differences between SUD and T2D

How can we explain the discrepancies between SUD and T2D in chronic illness treatment despite their mutual chronic illness definition? Historical analyses show that SUD has been perceived as a moral disease.^38^, ^39^ Contemporary studies also suggest that SUD is regarded as a mental disorder associated with questions of morality.^40^ For example, SUD is constituted by compromised autonomy due to recurrent self‐injuring behaviour.^41^ However, the T2D guideline also contains moral elements. T2D has been termed a ‘lifestyle’ disease caused by the patients, which is associated with moral attribution^42^, ^43^ in the sense of morally ‘good’ or ‘bad’ lifestyle choices.^42^, ^44^ What may distinguish SUD and T2D, both historically and currently, is that SUD is perceived as a mental disorder and associated with criminal behaviour.^45^, ^46^ Consequently, SUD is a condition treated by the health services and by legal institutions. From a medical perspective, SUD is viewed as a mental disorder in need of treatment, while from a juridical perspective, SUD is perceived as an illegal activity in need of correction. SUD's illegality makes several additional moral concepts relevant to understanding it, compared to T2D. The concepts of misuse, illegality, illicitness, addiction and abuse, all have legal connotations.^47^, ^48^

Does this ambiguity exclude the possibility of SUD being treated as a chronic illness? No. Does it explain why SUD practice follows an acute care model?^49^ No. It does, however, illuminate an ambiguity in SUD treatment in terms of institutional responsibility and in the broader understanding of SUD and its treatment. Following our line of argument above, SUD's association with criminalization may also have implications for global policymaking.^50^ This association may lead to discussion of whether SUD is a mental disorder, criminal behaviour or both. If drug policy is understood, as Benoit^51^ argues, as being grounded in institutions and politics, then institutions probably influence drug policymaking in different ways. Arguably, health care services and penal systems have different *raisons d'être* and, as a result, they produce different consequences. Furthermore, they have distinct ways of approaching the drug user. On the one hand, when conceptualized as a mental disorder, SUD is consigned to medical discourse and the mental healthcare services. On the other, when conceptualized as a criminal offence, SUD is regarded as belonging to the juridical discourse and as an issue to be treated by the criminal justice system. Benoit^51^ states that the change in drug policy between the United States and Canada after the 1980s was due to changes in institutions. Ultimately, these institutional changes led to law enforcement receiving more funding than health care services in the United States, while the opposite happened in Canada. As a result, Benoit^52^ suggests that SUD treatment in the United States received less funding than what was the case in Canada. It is difficult, however, to assess whether this retrenchment impeded SUD treatment development in the United States as compared to Canada.

Historically, Norwegian treatment guidelines have been associated with so‐called moral treatments of dependence that often consisted of labour activities over longer periods to discipline the substance abuser.^53^ However, drug and alcohol treatment have seen new trajectories.^53^ For example, moderate alcohol use was deemed acceptable in Norwegian culture (since the 1930s), while drug use was not (e.g., the use of cannabis, heroin or other drugs were criminalised). Drug policy was put on the political agenda in the 1960s, mainly focusing on zero tolerance in the sense that the use and possession of illegal substances without the prescription by a doctor were criminalised.^53^ This stance resonates with 20th century US drug policies.^54^, ^55^ In the 1990s, Norwegian policies included measures to reduce the harmful effects of substance abuse. However, Norwegian drug policy has been characterized as repressive compared to some other European countries.^56^ Contemporary Norwegian drug policy is based on the prohibition of use and possession of illegal substances. Notably, a policy‐revision transferring the institutional responsibility from the judicial system to the health care services was suggested in 2018.^57^ This policy has, however, not been put into force. Ødegård^58^ observes a tension within Norwegian drug policies. On the one hand, drug use is punished juridically. On the other, health care policies are in place that offer substance abusers free syringes and supervised injections. Another feature of Norwegian drug policy is that it aims to be evidence‐based. The Norwegian Institute of Public Health (NIPH) is responsible of gathering the evidence. Moreover, the NIPH is associated with the European Monitoring Centre for Drugs and Drugs Addiction (EMCDDA).^59^ Among other things, the NIPH is obligated to deliver annual reports on drug statistics, politics and measures to the EMCDDA. The EMCDDA provides the UN with research purportedly enabling them to make decisions about drug policy based on objective and verified facts rather than ideology or moral or value judgements.^60^ The relationship between the NIPH and the EMCDDA may implicitly affect Norwegian drug policy in the sense that the NIPH have similar objectives. However, the EMCDDs stance presupposes that research is not based on value judgements, which is not an unproblematic assumption.

### 5.2 SUD long‐term treatment and the normative foundation of drug policy

We have investigated guidelines developed in Norway. Hence, our findings may not be applicable to other countries. However, international research suggests that SUD treatment in general has been short termed and based on acute care.^61^, ^62^ While acknowledging that treatment practice appears to be increasing its use of continuing care and to consider SUD to be a chronic illness, McKay^63^ states that ‘the development of evidence‐based clinical practice guidelines to facilitate wider implementation of effective continuing care would be a major advance for the field’. This statement suggests that continuing care is not presently implemented in US SUD treatment guidelines. SUD guidelines appear to focus less on what to do after 3–6 months of treatment.^61^, ^63^ This observation indicates a general tendency to disregard long‐term recovery extending to 12 months in the SUD field.

Drug ‘problems’ ‘are never exogenous to (outside of) social and political practices*’*.^64^ This statement by Bacchi^64^ underscores that drug ‘problems’ and related practices are tied to the social and historical conditions of the possibility of knowledge.^65^ The historical conditions of possibility are tied to more or less explicit normative presuppositions. The selection of evidence and clinicians' and patients' experiences are evaluative and value‐laden judgements. SUD research propositions are not solely factual, descriptive propositions, but inherently normative.^66^ Different treatment interventions and disorder understandings rest on normative presuppositions: the claim that SUD is a brain disease is based on a different set of values than is the claim that it is a moral failing.^67^, ^68^ In this regard, social, political and historical conditions upon which the understanding of SUD is contingent are interwoven with the normative presumptions of the clinician, patient, treatment guidelines and policies. Drug policy reflects, among other things, decision‐making about drug control, drug treatment, prevention and harm reduction.^69^ SUD treatment guidelines also represent decision‐making as to what qualifies as evidence for a given recommendation: clinicians' tacit, practical and contextual knowledge and patients preferences, for instance, are not grounded in empirical findings.^70^ This decision‐making process is inseparable from values since values are prescriptive, or action‐guiding.^71^ Consequently, global drug policies and treatment guidelines carry normative presumptions about how SUD is understood and how it should be treated that may impede or limit the development of continuing care.

However, as SUD fulfils the criteria for chronic illness, drug policymaking should consider it as such. Although some SUD patients quit drugs earlier than others, they often struggle with dependence for several years before remission.^34^ Moreover, SUD is often a multicomorbid disorder, that is containing additional mental and somatic disorders, and SUD multicomorbidity is associated with other chronic illnesses.^72^ Whereas SUD patients often have social challenges, such as socioeconomic deprivation, legal issues and housing problems,^73^ and suffer from multicomorbidity, T2D patients do not seem to exhibit a similar degree of severity. This complex intersectionality suggests that SUD requires well‐coordinated and long‐term multiagency approaches. Lest SUD is acknowledged as a chronic illness, drug policymaking may overlook the importance of including scheduled long‐term follow‐up with systematic assessments to provide continuing care,^35^, ^74^ thus leading to suboptimal treatment and reducing the chances of long‐term recovery. Furthermore, when neglecting to see SUD as a chronic illness, the fact that SUD is a long‐term and recurrent disorder (for some but not all people) is disregarded.^61^ A chronic disorder does not mean that it is lifelong, but that it persists for an extended period.^75^ According to WHO's and others' definitions of chronic illness, a long period consists of at least 3–12 months. Moreover, the term ‘chronic’ denotes conditions that last for a long period and are resistant to cure,^75^ but ‘chronic’ does not mean that a condition is incurable. SUD fulfils the criteria for chronic illness, and research indicates that treatment follow‐up should extend 12 months, which suggests that a precondition for drug policymaking should be the recognition of SUD as a chronic illness.

### 5.3 SUD research consists of more short‐term than long‐term research

One possible explanation for the absence of continuing care in treatment guidelines and policies may be found in SUD research: SUD research focuses more on short‐term than on long‐term follow‐up and more on substance reduction measures than on recovery measures.^76^, ^77^, ^78^ SUD science's priority of short‐term follow‐up and substance reduction measures are value‐based. For example, by primarily using substance use reduction measures when assessing treatment outcomes, SUD recovery becomes conflated with reduction in substance use. However, this approach neglects the fact that a reduction in substance use also involves life‐style changes that improve one's ability to refrain from drug use. Furthermore, the emphasis on short‐term follow‐up suggests that SUD is treated as a short‐term disorder. Additionally, few studies have evaluated interventions with a scheduled duration of more than 6 months.^79^ Taken together, these research approaches come to give the impression that what is essential to successful treatment outcomes occurs within a short period (i.e., less than 2 years).^76^ In this regard, the knowledge base on which continuing care treatment guidelines are based is smaller than the knowledge base for acute care. In turn, this lack of knowledge may impede the progression of continuing care treatment guidelines.

Another possible explanation for the absence of continuing care in treatment guidelines and policies is the contradictory understanding of SUD as a chronic disorder or as ‘maturing out’. For example, Heyman^34^ has shown that dependence has the highest remission rate among psychiatric disorders and that those who remit are more likely to stay remitted than to relapse. However, Heyman^34^ argues that it is easy to find studies that support the view of SUD as a chronic disorder or SUD as a time‐limited disorder. Therefore, Heyman^34^ concludes that ‘in principle, they are simply the opposite ends of a distribution of time‐spent‐addicted durations: Some drug users quit early; some quit late*’*. However, this contrary view of dependence may influence policymaking, as the views are neither integrated nor seen ‘as two sides of the same coin’. Consequently, the lack of integration between opposing views of dependence, as either ‘chronic’ or ‘maturing out’, and the small knowledge base for long‐term SUD recovery may have implications for global policymaking.

## 6 CONCLUSION

We have found that T2D and SUD both correspond to WHO's definition of chronic illness. However, we argue that only T2D has been treated as a chronic illness in practice. The SUD guideline is nebulous compared to the T2D guideline. In the T2D guideline, the duration of follow‐up, the responsibility for treatment, responsibilities of the municipality, and expectations of patients are more explicit, presumably contributing to more straightforward implementation in practice. We suggest that this discrepancy in the treatment of these two illnesses may be a consequence of SUD's equivocal relationship to health and legal institutions. SUD's equivocal connection to health and legal institutions may create confusion about treatment responsibilities and whether SUD is a mental disorder or deviant behaviour. Furthermore, SUD research consists of more studies of short‐term follow‐up than of long‐term follow‐up. Thus, SUD science has a limited knowledge base as regards long‐term recovery, which may create limitations in terms of informing continuing care treatment guidelines. The attitude towards SUD as either a disorder or a deviant behaviour may, along with the scarcity of long‐term recovery knowledge, contribute to uncertainty about what knowledge should inform continuing care guidelines and may impede the progression of SUD treatment guidelines. Whether or not our arguments can be said to depict the whole causal picture, the consequence remains that the delivery of treatment services is inequitable: Equivalent cases are treated differently.

## CONFLICTS OF INTEREST

The authors declare no conflicts of interest.

## Data Availability

No data are available.
